# Assessment of efficacy and safety of SlimSEB^®^ for weight reduction in overweight and obese subjects at risk of type 2 diabetes mellitus: a prospective, randomized, double-blind, parallel-group, controlled, interventional clinical study

**DOI:** 10.3389/fnut.2026.1956865

**Published:** 2026-10-21

**Authors:** Abhijit Rathi, Abhijeet B. Muley, V. L. Rathi

**Affiliations:** Human Nutrition Department, Advanced Enzymes Technologies Ltd., Thane, India

**Keywords:** glucose and insulin, inflammation, lipid metabolism, overweight and obesity, SlimSEB^®^, weight management

## Abstract

**Objective:**

The current clinical study intended to explore the clinical efficacy and safety of SlimSEB^®^ for weight reduction in overweight and obese subjects with the risk of type 2 diabetes mellitus.

**Methodology:**

Eighty-seven subjects (43 overweight and 44 obese) were enrolled in this prospective, interventional, randomized, double-blind, parallel, controlled, clinical study. Subjects were randomized for daily administration of SlimSEB^®^ or placebo for 84 days. The change in anthropometric parameters (body weight, BMI and waist circumference), glucose and insulin related factors (fasting plasma glucose, fasting insulin, hemoglobin A1c, homeostatic model assessment for insulin resistance, homeostasis model assessment of β-cell function, disposition index, and quantitative insulin sensitivity check index), and lipid profile and biomarkers (total cholesterol, LDL, HDL, triglycerides, and CRP) were assessed. Additional outcomes included changes in visual analogue scale and tolerance to SlimSEB^®^, and the incidences of adverse events (AEs) and serious adverse events (SAEs).

**Results:**

The oral supplementation of SlimSEB^®^ was associated with greater reductions in anthropometric, glycemic, lipid and metabolic parameters than placebo. The body weight, BMI and waist circumference showed significantly greater reductions with SlimSEB® than placebo, with adjusted least square mean differences of −2.60 kg and −4.51 kg (*p* = 0.0045, *p* < 0.0001), −0.92 kg/m² and −1.80 kg/m² (*p* = 0.0063, p < 0.0001), and −3.86 cm and −11.03 cm (*p* = 0.0169, *p* = 0.0014) in overweight and obese subjects, respectively. Significant adjusted improvements were also observed with SlimSEB^®^ for fasting plasma glucose (−0.45 mmol/L, −0.45 mmol/L; *p* = 0.0124 and *p* = 0.0068), HbA1c (−0.65% and −0.67%; *p* = 0.0199 and *p* = 0.003), HOMA-IR (−0.47, −0.69; *p* = 0.0205 and *p* = 0.002) in overweight and obese subjects. The lipid parameter analyses showed significant adjusted reductions in total cholesterol by 14.21 and 16% with SlimSEB^®^ in overweight and obese subjects, compared with 3.85% reduction and 8.65% increase in placebo (*p* = 0.0089 and *p* < 0.0006). The LDL cholesterol also decreased in overweight and obese subjects by 11.61 and 14.63% over 0.88% reduction and 13.39% increase in placebo (*p* = 0.0154 and *p* = 0.0002). CRP levels decreased by 13.6 and 17.3% with SlimSEB^®^ in overweight and obese subjects, respectively, with a significant inter-group difference in obese subjects (*p* = 0.0001). SlimSEB^®^ was well tolerated at the studied dose, with eight mild AEs reported, none of which was considered related to SlimSEB^®^, and no SAEs were reported.

**Conclusion:**

Daily intake of SlimSEB^®^ resulted in a reduction in body weight, BMI and waist circumference, and showed beneficial changes in selected glucose and lipid parameters, and promote a healthy inflammatory response. These findings suggest that SlimSEB^®^ may support weight management and metabolic health. Further well-designed clinical trials involving larger study populations and longer intervention periods are needed to understand the underlying mechanisms of action.

**Clinical trial registration:**

The current investigation was registered in the Clinical Trial Registry, India (CTRI registration number: CTRI/2023/11/059798), and registered on 14/11/2023 (https://ctri.nic.in/Clinicaltrials/pmaindet2.php?EncHid=OTM3Mjk=&Enc=&userName=).

## 1 Introduction

Globally, overweight and obesity has become a serious public health concerns among adults and older population, with their prevalence increasing at an alarming rate (1, 2). The global number of individuals living with obesity rose drastically to 641 million in 2014 from 105 million in 1975 (3). According to World Health Organization (WHO), in 2022, 43% of adults aged 18 years and older were overweight, while 16% were living with obesity (4). Recent studies have consistently reported similar increasing trends in the prevalence of overweight and obesity worldwide (5). WHO identifies body mass index (BMI) as the most appropriate and widely used indicator for classifying overweight and obesity. Individuals with a BMI of ≥25 kg/m^2^ are classified as overweight, whereas those with a BMI of ≥30 kg/m^2^ are classified as obese (6). Overweight is characterized by the excessive accumulation of body fat, while obesity is a chronic and complex disease in which excess adiposity adversely affects health, reduces quality of life, and imposes substantial economic burdens on individuals and healthcare systems (7, 8). Persistent overweight or obesity could pose serious life-threatening complications and impair other physiological systems (9–11). Both conditions are correlated with a low to mild grade, and a chronic state of inflammation, and are associated with a higher risk of morbidity and mortality than underweight. Obesity has consequently become the fifth leading cause of death (1, 11–13). Furthermore, a recent global burden of disease study estimated that elevated BMI was associated with approximately 2.3 million deaths among men and 2.4 million deaths among women, globally (14).

Overweight and obesity are primarily developed due to an imbalance between energy expenditure and calorie intake, which may arise from multiple contributing factors (11, 15). Effective weight management requires a multidisciplinary approach that includes lifestyle modification, structured self-management education, reduction of obesity-related health risk factors, improved dietary habits and adherence, pharmacotherapy, dietary supplementation, regular physical activity, stress management, behavioural therapy, and, where appropriate, bariatric surgery (15–20). Although each of these interventions may contribute to weight reduction, no single approach consistently ensures optimal efficacy or sufficient caloric deficit because of variations in individual responses. Therefore, a combination of therapeutic strategies is often necessary to achieve and sustain clinically meaningful weight loss (21). In recent years, nutraceuticals (22), enzyme supplements (23), and probiotics (24) have gained increasing attention as complementary or adjunctive interventions, either as alternatives to conventional therapies or in combination with them.

Various probiotics and their combinations are explored for assessment of efficacy in weight management and associated parameters, and thoroughly covered in a meta-analysis of randomised control trials (24). The list of probiotics includes *Lactobacillus* sp., *Bifidobacterium* sp., *Lactococcus* sp., *Enterococcus* sp., *Streptococcus* sp., and many others. Campbell et al. has reported the effectiveness of *Bacillus* spp. in significantly reducing the triglyceride levels of mild to moderate hypertriglyceridemia patients (25). Probiotics colonize the intestine and produce metabolites that supports maintenance of intestinal homeostasis, help restore gut microbiome, replenish beneficial functional activities, reduce pH, inhibit the growth of harmful opportunistic pathogens, and supports the intestinal mucosa in recovering from injuries (26–28). These may also support digestion of food and help to lower the glucose and lipid levels (28, 29). A recent systematic review and meta-analysis of randomized controlled trials revealed the contribution of probiotics in decreasing the size of adipocytes by reducing absorption of fatty acids and improving the expression of genes involved in the oxidation of fatty acids (30). Further, a review article has shed light on the positive effects and role of probiotics in nutrient absorption and energy regulation (31).

Exogenous digestive enzymes can act synergistically with endogenous enzymes to hydrolyze dietary macronutrients, thereby improving digestion and enhancing nutrient availability and absorption (32, 33). During the in-vitro gastric phase of digestion, SucroSEB™ (a model enzyme for alternansucrase) demonstrated a significant capacity to convert sucrose into dietary fibers in the presence of various acceptor sugars (34). These dietary fibers may play an important role in promoting a healthy gut microbiota while providing additional physiological benefits (35). Furthermore, 12-week supplementation with transglucosidase has been associated with improvements in blood glucose, HbA1c, and insulin levels (36), as well as favorable alterations in the gut microbiome (37), bowel motility, and fecal short-chain fatty acid profiles in individuals with T2DM (38). Similarly, supplementation with enzyme blends and probiotics in combination with whey or pea protein has been reported to enhance the rate of amino acid absorption and induce modest but beneficial changes in the gut microbiome, respectively (39, 40).

In addition to enzymes and probiotics, numerous herbal-derived products have been investigated for their potential efficacy in managing overweight and obesity. These products are often regarded as a first-line approach for promoting health by preventing obesity-related diseases and associated complications (41). Herbal products are rich sources of bioactive phytochemicals that exhibit anti-inflammatory, antioxidant, immunomodulatory, antimicrobial, and antihypertensive properties, thereby contributing to weight management and overall metabolic health (42). The anti-obesity effects of herbal medicines are mediated through several mechanisms, including (i) suppression of appetite, leading to reduced energy intake; (ii) stimulation of thermogenesis and enhancement of energy metabolism; (iii) inhibition of pancreatic lipase activity, resulting in decreased fat absorption; (iv) promotion of lipolysis; and (v) inhibition of lipogenesis (43).

While individual approaches involving probiotics, enzymes, or herbal extracts may have a positive impact, the efficacy of a product containing a combination of all these ingredients for weight reduction and other related parameters has not been well-documented in the literature. A recent pilot study evaluating SlimSEB^®^ as an adjunct to GLP-1 therapy in patients with T2DM suggested potential improvements in health-related quality of life and weight regulation, highlighting the potential value of complementary nutritional interventions alongside pharmacotherapy (44). However, the study was conducted exclusively in patients with T2DM and did not comprehensively evaluate glycemic and insulin-related parameters, hematological indices, or biochemical biomarkers, limiting the understanding of its broader metabolic effect. Overall, the limited availability of well-designed human clinical studies on weight management underscores the need for robust randomized clinical trials evaluating the efficacy and safety of multi-ingredient formulations, including probiotics, digestive enzymes, and herbal extracts, for sustainable weight management. Considering the excellent potential of probiotics, digestive enzymes, and nutraceuticals in managing weight and other disorders in individuals with overweight and obesity, the development of a product combining these ingredients (SlimSEB^®^) warrants further exploration. Accordingly, a randomized, double-blind, parallel, and placebo controlled clinical trial was performed to assess the safety and efficacy of SlimSEB^®^ in subjects with weight related issues caused by overweight and obesity. To the best of author’s information, this is the first study wherein a SlimSEB^®^ is explored for weight reduction in subjects with the risk of T2DM. Furthermore, the inclusion of both overweight and obese subjects makes the investigation unique. The assessment of changes in anthropometric measurements, glucose and insulin related factors, and lipid profile and inflammatory biomarkers was performed. In addition, the safety and tolerability of the product were determined by assessing the changes in vital physical signs and haematological parameters, along with clinical signs and recording the adverse events (AEs) and severe adverse events (SAEs).

## 2 Materials and methods

### 2.1 Materials

#### 2.1.1 Investigational product

The investigational product (IP), was formulated as oral capsules in a desired strength, required compliance, quality and standard was provided by Specialty Enzymes and Probiotics, Chino, USA, whereas the placebo capsules consisted of only maltodextrin. SlimSEB^®^ is a blend of probiotics—1.5 billion CFU (*Alkalihalobacillus clausii* 088AE, *Bacillus subtilis* PLSSC, *Heyndrickxia coagulans* LBSC, *Lactiplantibacillus plantarum* 022AE, and *Lactobacillus gaserri*), enzymes (SucroSEB 120 U, amylase-800 DU, β-amylase-200 DU, proteases-2000 HUT, lactases-100 ALU, α-galactosidase-20 GaL U, chitosanase-20 mg, glucose oxidase-400 GOU, and HemiSEB-200 XU), and 95.5 mg other ingredients (Mulberry leaf extracts, green tea, cinnamon powder, and chromium picolinate). The packaging, physical appearance, size, weight, color, and labeling of the IP and placebo were identical, making the two products indistinguishable. Sample identification, randomization, and treatment allocation were managed using coded batch numbers to ensure blinding throughout the study.

### 2.2 Ethics and informed consent

The committees of (i) Medstar speciality hospital ethics committee of Medstar Speciality Hospital, Bangalore [ECR/1324/Inst/KA/2019], (ii) Muktai hospital institutional ethics committee of Lifecare hospital, Nashik [ECR/251/Inst/Maha/2013/RR-19], and (iii) Swasthyam ethics committee of Swasthyam Superspeciality Hospital, Nagpur [ECR/1558/Inst/MH/2021] reviewed, recommended changes in the study protocol (if required) and provided approval to the designed study protocol before the initiation of actual clinical study. The said clinical study was registered in the Clinical Trial Registry, India (CTRI registration number: CTRI/2023/11/059798, and registered on 14/11/2023) before the enrolment of any subjects for the mentioned clinical trial (https://ctri.nic.in/Clinicaltrials/pmaindet2.php?EncHid=OTM3Mjk=&Enc=&userName=). The design of protocol for clinical study was done by pertaining the pre-requisites of ICH - GCP E6 (R2), Declaration of Helsinki (2013) (45), New Drugs and Clinical Trial Rules (2019) (46) and ICMR Guidelines for Biomedical and Health Research involving Human Participants (2017) (47), and guidelines affirmed by FSSAI. All the methods were performed in accordance with the relevant guidelines and regulations. The finally approved protocol was practiced strictly, and no further changes or deviations were made during the entire period of the investigation. All the participants who were enrolled in the investigation were made aware and correctly informed about the particulars and details of the clinical study. All the necessary details and information associated with the said clinical investigation was described, both orally and in written format, in the vernacular (suitable, understandable and familiar to them) as well as English. The participant/s submitted a written informed consent after a clear understanding of each bit of information and details were explained to them. This information included related objectives, probable health benefits, and risks of the intervention product. The study was performed at Medstar Speciality Hospital, Bangalore, Lifecare Hospital, Nashik, and Swasthyam Superspeciality Hospital, Nagpur.

### 2.3 Clinical study design and selection of study subjects

The current prospective and interventional clinical study is a randomized, double-blind, parallel, and placebo controlled trial. The clinical study possessed four investigation arms (two SlimSEB^®^ study arms and two placebo arms, which included overweight and obese subjects), a total of five visits, and a study duration of 98 days. The total interventional period with the IP was 84 days with efficacy analysis at start of treatment (Day 0), intermediate analysis in mid of study (day 42), final analysis at end of treatment (day 84), and a final follow up after a 2 weeks wash out period (day 98). The safety and tolerability of the product were assessed during each study visit. The five visits of the study were: Visit 1 - Baseline visit, subject screening and randomization; Visit 2 to 4 - Visits to clinical sites which included patient enrolment, study of baseline parameters, beginning of intervention, intermediate analysis, final analysis, and end of treatment with respective safety assessment; Visit 5 - End of clinical study with assessment of safety and tolerability of products. The current designed study was conducted on the registered and included subjects recruited as per a pre-defined inclusion and exclusion criteria.

#### 2.3.1 Inclusion criteria

Subjects meeting the following inclusion criteria were deemed eligible: (1) Male/female participant of ≥30 and ≤65 years of age. (2) Participant who can and is willing to provide written informed consent. (3) Participants meeting WHO defined criteria for overweight (BMI, 26–29.9 kg/m^2^) or obese (BMI ≥ 30 kg/m^2^). (4) Participant with the high risk for T2DM (fasting glucose) for impaired fasting glycaemia [IFG, 6.1–7.0 mmol/L (110–125 mg/dL)]. (5) Participant/s legally acceptable representative understands and can comply with clinical trial protocol requirements.

#### 2.3.2 Exclusion criteria

Subjects meeting the following exclusion criteria were deemed non-eligible: (1) Participants ≤30 or ≥65 years of age. (2) Participants who had T2DM [(fasting glucose) ≥ 7.0 mmol/L (≥126 mg/dL)]. (3) Participants who had other comorbidities, metabolic syndrome, or non-alcoholic steatosis. (4) Participants who were currently undergoing any weight loss treatment through diet restrictions or medications and were not willing to refrain from that regimen for at least 1 month prior to enrollment. (5) Participants who had undergone aggressive diet attempts within the last 3 months or were addicted to alcohol or drugs. (6) Participants with a current or historical treatment that may have caused significant weight gain. (7) Participants with eating disorders, e.g., anorexia nervosa, bulimia nervosa, and binge-eating disorder. (8) Participants who had a weight change of ≥5 kg in past 3 months. (9) Pregnant and/or nursing woman. (10) Participant with any clinically significant illness. (11) Participants contracted with COVID-19 infection. (12) Participants with a history or presence of coronary, renal, pulmonary, and thyroid disease. (13) Participants who were active or had a history of inflammatory bowel disease. (14) Participants who had undergone any abdominal surgery, except for hernia repair or appendectomy. (15) Participants who were receiving medication to lower serum cholesterol levels. (16) Participants who had used probiotic products within 6 weeks prior to screening in the current trial or were currently receiving other products. (17) Participants who had undergone treatment with antibiotics, systemic steroids, or antipsychotic therapy within 14 days prior to screening. (18) Participants with a history of hypersensitivity to any of the components of the investigational products or excipients used in the formulation of the IP. (19) Participants participated in another trial involving an investigational product within 1 month prior to this trial. (20) Participants who, in the judgment of the investigator, were unlikely to comply with the requirements of the protocol.

### 2.4 Study procedure

#### 2.4.1 Subjects, randomization, intervention and procedures

A total of 87 subjects were enrolled for the said clinical investigation following the pre-defined selection criteria, and each subject was assigned a unique subject identification code. The sample size was calculated assuming a two-arm, parallel-group study with a 1:1 allocation ratio, 90% power, and a two-sided significance level of 5%. For overweight participants, an expected minimum between-group difference of 2.3 kg and a standard deviation of 2.45 kg were assumed, whereas for obese participants, an expected difference of 3.5 kg and a standard deviation of 3.75 kg were assumed. The sample-size calculation was performed using the following formula:


$$n=\frac{2{\left(z_{1-\alpha /2}+z_{\beta }\right)}^{2}\sigma^{2}}{\varepsilon^{2}}$$

Where, 𝑛 is the required sample size per group, σ is the standard deviation, 𝑍_1−𝛼/2_ is the standard normal deviate corresponding to a two-sided significance level of 5% (1.96), 𝑍_1−𝛽_ is the standard normal deviate corresponding to 80% power (0.84), and 𝜀 is the expected difference in means, representing the difference in means (48).

Based on these assumptions, the calculated sample size was approximately 18 participants per treatment group within each BMI stratum. Thus, for the two treatment groups across the two BMI strata (overweight and obese), the initial sample size was 72 participants (18 × 2 treatment groups × 2 BMI strata). Allowing for a 15% dropout rate, the sample size was increased to approximately 21 participants per treatment group within each BMI stratum, resulting in a total target sample size of 84 participants (21 × 2 treatment groups × 2 BMI strata). Accordingly, 87 participants were enrolled in the clinical investigation.

Importantly, the overweight and obese strata did not constitute separate randomized trial arms. The randomization remained 1:1 to SlimSEB^®^ or placebo, and the BMI categories were considered subgroup factors within this randomized comparison. Thus, the sample-size planning by BMI stratum does not alter the primary randomized treatment comparison.

The subjects were randomized into SlimSEB^®^ intervention group (*N* = 44) and placebo group (*N* = 43) in 1:1 ratio using a block randomization design (randomization with block size of 6). The randomization sequence was generated by using simple randomization technique method post preparation of a master randomization list prepared by a biostatistician and shared with the designated unblinding person of the study sponsor. Allocation concealment was maintained using Independent Sequence Generation. The unblinding person labelled the bottles with the respective randomization numbers. Based on these labels, the site staff carried out the randomization as per the assigned numbers. The packaging, physical appearance, size, weight, colour, and labelling of both the products, IP (SlimSEB^®^) and Placebo, were exactly identical and making it difficult to differentiate. The samples, identification, randomization and treatment differentiation of both products were performed based on of coded batch numbers. The randomized subjects were further allocated to the overweight (*N* = 23 Intervention group and *N* = 20 Placebo group) and obese groups (*N* = 21 Intervention group and *N* = 23 placebo group), respectively. Both the subjects, overweight and obese, in the intervention arm were asked to orally consume two capsules containing IP with drinking water twice a day (afternoon and evening) for 84 consecutive days, 15–20 min before meals. On the other hand, both the subjects (overweight and obese) in the placebo group were only on maltodextrin with the same dosing schedule. The details of the study design, which include distribution of subjects, allocation of treatment, and other procedures followed are presented in the CONSORT flow diagram (Figure 1), and study schedule (Table 1). All the enrolled subjects, clinicians, physicians, investigators and officers associated with the clinical investigation were kept blinded until the completion of the trial.

**Figure 1:**
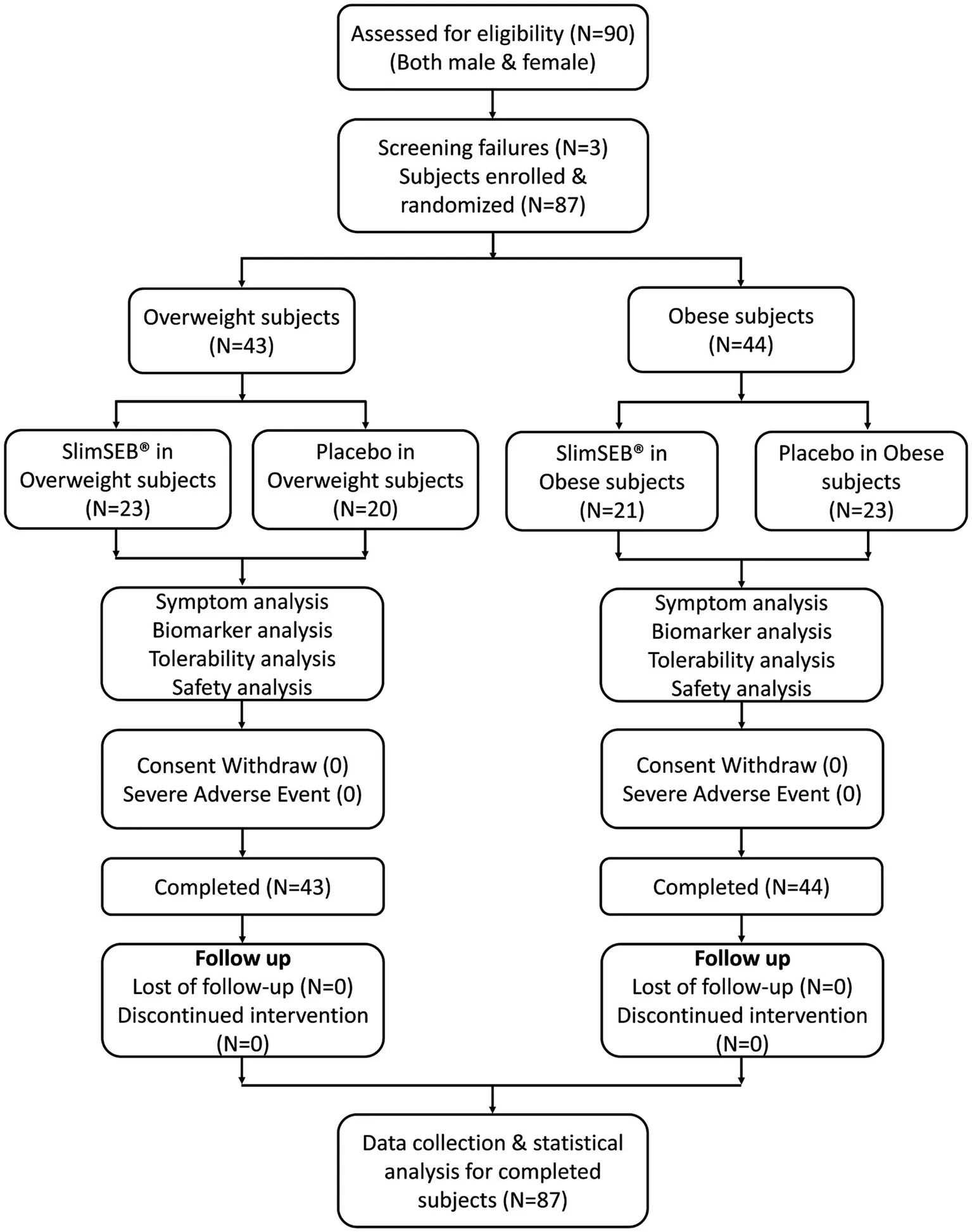
CONSORT flow chart of study participants.

**Table 1:** The schematic representation of study procedures schedule.

Activities	Visit 1	Visit 2	Visit 3	Visit 4	Visit 5
	Screening/Baseline visit (Day −2)	Enrolment visit (Day 0)	Intermediate analysis (Day 42)	End of treatment (Day 84)	End of study (Day 98)
Informed consent	✓				
Demography	✓				
Anthropometry	✓		✓	✓	
Medical/Surgical History along with prior medications	✓				
Physical Examinations	✓	✓	✓	✓	
Vital signs	✓	✓	✓	✓	
Inclusion/exclusion criteria	✓	✓			
Urine pregnancy test*		✓#			
Clinical lab tests	✓			✓	
Randomization		✓			
Biomarker test	✓		✓	✓	
Insulin sensitivity parameters tests	✓		✓	✓	
Tolerability Assessment				✓	
Study drug dispensing		✓	✓		
Study drug accountability			✓	✓	
Concomitant medication	✓	✓	✓	✓	✓
Adverse event	✓	✓	✓	✓	✓

*Urine pregnancy test was done for females of childbearing potential only. #Eligibility reconfirmation window period of ±2 days is applicable for all visits except Visit 1.

The clinical coordinators closely monitored the intervention and conducted periodic follow-ups with the enrolled subjects. During these follow-ups, a structured set of questionnaires on items related to IP consumption was also included to monitor or assess the participants’ compliance with the intervention protocol. The un-blinding was performed after the execution of the post-clinical phase of the investigation. All the supportive medications and treatment(s), if needed, were prescribed and provided to the subjects by the clinical investigator or attending physician. The use of anti-microbial agents in combination with the IP was strictly avoided. The finally designed and approved protocol of the clinical study was austerely practiced, and no further deviations or amendments were incorporated after the approval of study. Lastly, during the entire course of study no assessments of intermediate data nor disclosure of recorded data was done, and the interim analysis of any of the data generated were not recommended nor practiced.

#### 2.4.2 Clinical evaluation and outcome measurements

The clinical investigator performed the assessment of physical and hematological parameters and eventually collected the data to determine the primary and secondary outcome measures at baseline and pre-defined time points. The assessment involved the determination of efficacy and safety indicators of subjects during course of the clinical study.

##### 2.4.2.1 Primary outcomes

The primary outcomes were set to assess the efficacy of SlimSEB^®^ for weight reduction in overweight and obese subjects at a risks of T2DM. This included determination of (1) Change in anthropometric measurements: (i) Body weight (kg), (ii) BMI (kg/m^2^), and (iii) Waist circumference (cm); (2) Change in the glucose and insulin related factors: (i) Fasting plasma glucose (FPG) (mmol/L), (ii) Fasting insulin (μIU/mL), (iii) Hemoglobin A1c (HbA1c) (%), (iv) Homeostatic model assessment for insulin resistance (HOMA-IR), (v) Homeostasis model assessment of β-cell function (HOMA-β), and (vi) Disposition index; (3) Change in lipid profile and biomarkers (mg/dL): (i) Total cholesterol, (ii) LDL, (iii) HDL, (iv) Triglycerides, and (v) CRP.

The HOMA-IR, HOMA-β, DI and QUICKI were calculated as


$$\text{HOMA}-\mathrm{IR}=\frac{\left[\text{Fasting insulin}\;(\frac{\mathrm{\mu IU}}{\mathrm{mL}})\times \text{Fasting glucose}\;(\frac{\text{mmol}}{L})\right]}{22.5}$$

$$\text{HOMA}-\beta =\frac{\left[20\times \text{Fasting insulin}\;(\frac{\mathrm{\mu IU}}{\mathrm{mL}})\right]}{\left[\text{Fasting glucose}\;(\frac{\text{mmol}}{L})-3.5\right]}$$

$$\mathrm{DI}=\frac{450}{\left[{\left(\mathrm{FPG}\right)}^{2}-(\mathrm{FPG}\times 3.5)\right]}$$

##### 2.4.2.2 Secondary outcomes

The secondary outcomes were set to assess the safety, tolerability and efficacy of SlimSEB^®^ on vital signs and clinical parameters in subjects with the risks of T2DM. This included determination of (1) Vital physical indicators: (i) Body temperature, (ii) Systolic blood pressure, (iii) Diastolic blood pressure, (iv) Pulse rate, (v) Respiration rate; (2) Vital haematological parameters: (i) Complete and differential blood count, platelets, and (ii) General biochemistry includes Alanine Aminotransferase (ALT), (Aspartate Aminotransferase) AST, serum creatinine, blood urea nitrogen (BUN), total albumin and globulin; (3) Tolerability assessment score of IPs at EOT; (4) Number of Treatment Emergent Adverse Event (TEAE) and Treatment Emergent Serious Adverse Event (TESAE). The AEs and SAEs occurring during the course of study were monitored and recorded managed appropriately from screening/baseline to end of study.

The tolerability of the products under study was evaluated by assessing the subjects for any side effects. The assessment was carried out at Visit-4 (Day 84), and evaluated with following scale [1 = Very good (no side effects), 2 = Good (insignificant side effects which do not cause serious problems to the patient), 3 = Satisfactory (side effects which affect the patient’s condition, but do not necessitate discontinuation of the formulation), 4 = Unsatisfactory (adverse side effect which significantly affects the patient’s condition and necessitates discontinuation of the formulation), and 5 = Highly unsatisfactory (adverse side effect which necessitates discontinuation of the formulation and use of additional clinical measures)].

### 2.5 Statistical analysis

All the data sets generated were primarily processed by using Microsoft Excel 2016, and the findings of all the experiments are presented as mean±standard deviation. The data was analyzed with a 95% confidence interval (significance level of 5%), and statistical analysis was performed using SAS^®^ software, version 9.1 with statistical analysis plan. The Kolmogorov–Smirnov test was used to test the normal distribution of data. The intra-group (within group) and inter-group (inter-groups) difference were assessed using “*t*-Test: paired Two Sample for Means” and “independent *t*-Test: Assuming unequal variance.” Further, the differences between the adjusted group means were represented calculated with Least Squares Mean Difference and represented as LS mean difference (SE), and the respective inter-group *p*-values were calculated using the “ANCOVA model with change from baseline as the dependent variable and visit. The *p* ≤ 0.05 was considered statistically significant otherwise specified. The efficacy and safety endpoints of all the 87 enrolled subjects were analysed with precision of 95% confidence interval (CI) for intention to treat (ITT) and PP population.

## 3 Results

The first subject was enrolled on November 29, 2023, and recruitment commenced on December 18, 2023. The last subject was enrolled on May 28, 2024, and the study was completed on September 5, 2024. Overall, participant recruitment was conducted from December 18, 2023, to May 28, 2024. The study was performed at three sites, and all the 87-intention to treat (ITT) subjects completed the study and there were no dropouts. Hence, all 87-ITT subjects were considered as per protocol (PP) population subjects for endpoints evaluation.

### 3.1 Characteristics of participants

The flow chart of participants enrolled in the study is presented in Figure 1. Altogether, 90 individual subjects with overweight and obesity were screened, found eligible, and also provided written informed consent for active participation in the clinical investigation. Amongst the 90-screened subjects, 3 were not eligible after following the exclusion criteria or not meeting the inclusion criteria. A total 87 subjects meeting the pre-defined criteria were deemed eligible for the clinical study by the principal investigator. These subjects were randomized into the SlimSEB^®^ intervention group (*N* = 44) and placebo groups (*N* = 43) by the block randomization design. These randomized subjects were further allocated to the overweight (*N* = 23 Intervention group and *N* = 20 Placebo group) and obese groups (*N* = 21 Intervention group and *N* = 23 placebo group), based on the BMI, respectively. All the 87-subjects completed the 84-day intervention period, and were included for the per-protocol set analysis and statistical analysis.

The demographic details of study subjects (both, overweight and obese) included in the clinical study at baseline are shown in Table 2. The baseline characteristics did not alter, and the difference between the two groups was statistically non-significant (*p* ≥ 0.05). The average age and BMI of overweight subjects in placebo and SlimSEB^®^ group were 44.80 ± 8.45 and 44.09 ± 8.54 years, and 28.01 ± 0.87 and 28.02 ± 0.92 kg/m^2^, respectively. Whereas the age and BMI of obese subjects in placebo and test group were 42.78 ± 7.76 and 43.33 ± 9.54 years, 32.36 ± 1.88 and 32.60 ± 1.89 kg/m^2^, respectively. The determination of study investigation regulations, safety protocols and efficacy assessments of each subject during every mentioned visit was carried out as per the pre-defined plan of the event by the principal investigator and the clinical trial co-ordination team (Table 1). The final conclusion of clinical investigation was drawn after the concluding and safety follow-up, i.e., visit 05 (Day 98) of the last registered and enrolled participant, and achievement of the required population size needed for the study.

**Table 2:** Demographic details and baseline characteristics of the subjects under study.

Parameter		Overweight subjects			Obese subjects		
		Placebo *N* = 20	SlimSEB^®^ *N* = 23	*p*-value (Inter group)*	Placebo *N* = 23	SlimSEB^®^ *N* = 21	*p*-value (Inter group)*
Gender	Male	12 (60.0%)	15 (65.20%)	0.7241^#^	16 (69.6%)	16 (76.20%)	0.6221^#^
	Female	8 (40.0%)	08 (34.8%)		7 (30.4%)	05 (23.80%)	
Age (years)		44.80 ± 8.45	44.09 ± 8.54	0.7850	42.78 ± 7.76	43.33 ± 9.54	0.8357
Body weight (kg)		74.3 ± 9.6	75.3 ± 7.2	0.7115	86.2 ± 7.0	86.5 ± 7.4	0.9095
Height (m)		1.63 ± 0.09	1.64 ± 0.08	0.6439	1.63 ± 0.07	1.63 ± 0.06	0.8214
BMI (kg/m^2^)		28.01 ± 0.87	28.02 ± 0.92	0.9577	32.36 ± 1.88	32.60 ± 1.89	0.6776
Waist circumference (cm)		97.7 ± 10.3	98.8 ± 7.8	0.7040	104.2 ± 7.3	104.4 ± 7.2	0.9282
Race		Asian (100.0%)	Asian (100.0%)	1.0000	Asian (100.0%)	Asian (100.0%)	1.0000
Physical activity	Mild	08 (40.00%)	08 (34.80%)	0.2879^#^	12 (52.20%)	12 (57.10%)	0.8856^#^
	Moderate	07 (35.00%)	10 (43.50%)		07 (30.40%)	05 (23.80%)	
	None	05 (25.00%)	05 (21.70%)		04 (17.40%)	04 (19.00%)	
Respiration rate (breathes/min)		17 ± 2	17 ± 2	0.7132	16 ± 2	17 ± 3	0.1072
Systolic blood pressure (mmHg)		126 ± 5	127 ± 6	0.4445	122 ± 6	128 ± 5	0.0006
Diastolic blood pressure (mmHg)		90 ± 6	85 ± 4	0.0018	87 ± 6	86 ± 5	0.4455
Pulse rate (Beats per min)		84 ± 6	88 ± 6	0.0255	92 ± 3	90 ± 2	0.0307
Body temperature (°F)		98.16 ± 0.27	98.14 ± 0.29	0.8466	98.19 ± 0.39	98.33 ± 0.27	0.1639
Fasting plasma glucose (mmol/L)		6.50 ± 0.22	6.52 ± 0.23	0.7557	6.43 ± 0.21	6.46 ± 0.20	0.6677
HbA1c (%)		6.30 ± 0.32	6.28 ± 0.34	0.8496	6.26 ± 0.31	6.40 ± 0.41	0.2117

*Inter group statistical analysis was performed using “*t*-test: two-sample assuming unequal variances.” ^#^Chi-square test was used for categorical variables.

### 3.2 Primary endpoint: efficacy evaluation

#### 3.2.1 Effects of SlimSEB^®^ supplementation on anthropometric parameters

The oral supplementation of SlimSEB^®^ caused a significant reduction in body weight and BMI in both overweight and obese subjects with the risks of T2DM post intervention of 84 days (Table 3). Significant inter-group differences were observed for body weight in both overweight and obese subjects. In overweight subjects, the body weight decreased by 2.4 ± 4.0 kg with SlimSEB^®^ compared with 0.1 ± 2.9 kg with placebo (inter-group *p* = 0.0316). This finding was further supported by ANCOVA, which showed a significant adjusted LS mean difference of −2.6 kg (95% CI: −4.343, −0.851; *p* = 0.0045). While in the obese subjects, the body weight decreased by 3.6 ± 1.7 kg with SlimSEB^®^ over an increase of 0.9 ± 3.6 kg with placebo. The difference in mean change between groups was highly statistically significant (*p* < 0.0001), with ANCOVA demonstrating an adjusted LS mean difference of −4.51 kg (95% CI: −5.984, −3.031; *p* < 0.0001). Intra-group reductions were significant with SlimSEB^®^ in both overweight (75.3 ± 7.2 kg to 72.9 ± 8.9 kg; 3.2%, *p* = 0.0083) and obese subjects (86.5 ± 7.4 kg at SOT to 82.8 ± 7.2 kg at EOT, 4.3%; *p* < 0.0001), whereas no significant changes were observed with placebo (74.3 ± 9.6 kg at SOT to 74.3 ± 11.1 kg at EOT; *p* = 0.9184, and 86.2 ± 7.0 kg at SOT to 87.1 ± 6.6 kg at EOT; *p* = 0.2419, respectively).

**Table 3:** Effect of SlimSEB^®^ supplementation on primary endpoints (anthropometric parameters, glucose and insulin related factors, and lipid and inflammatory response factors) in overweight and obese subjects.

Parameter	Group	SOT	EOT	Mean change (EOT-SOT)	*p-*value (Intra group)*	*p-*value (Inter group; independent *t*-test)**	LS mean difference (± SE)	95%CI for LS mean difference	*p-*value (Inter group; ANCOVA)^$^
Overweight subjects									
1. Anthropometric parameters									
Body weight (kg)	Placebo	74.3 ± 9.6	74.3 ± 11.1	−0.1 ± 2.9	0.9184	0.0316	−2.6 ± 0.87	(−4.343, −0.851)	0.0045*
	SlimSEB^®^	75.3 ± 7.2	72.9 ± 8.9	−2.4 ± 4.0	0.0083				
BMI (kg/m^2^)	Placebo	28.0 ± 0.9	27.9 ± 1.7	−0.1 ± 1.2	0.7757	0.0454	−0.92 ± 0.32	(−1.569, −0.275)	0.0063*
	SlimSEB^®^	28.0 ± 0.9	27.1 ± 2.0	−0.9 ± 1.5	0.0078				
Waist circumference (cm)	Placebo	97.7 ± 10.3	96.6 ± 12.4	−1.2 ± 6.3	0.4246	0.0777	−3.86 ± 1.56	(−6.995, −0.726)	0.0169*
	SlimSEB^®^	98.8 ± 7.8	94.1 ± 10.6	−4.7 ± 6.6	0.0024				
2. Glucose and Insulin related factors									
Fasting plasma glucose (mmol/L)	Placebo	6.50 ± 0.22	5.98 ± 0.80	−0.52 ± 0.86	0.0144	0.0555	−0.45 ± 0.17	(−0.804, −0.102)	0.0124*
	SlimSEB^®^	6.52 ± 0.23	5.56 ± 0.48	−0.96 ± 0.52	<0.0001				
Fasting Insulin (μIU/mL)	Placebo	13.07 ± 5.04	11.92 ± 5.84	−1.16 ± 2.52	0.5062	0.5062	−0.63 ± 0.57	(−1.76, 0.497)	0.2684
	SlimSEB^®^	12.94 ± 4.85	12.22 ± 4.84	−0.72 ± 1.57	0.0391				
HbA1c (%)	Placebo	6.3 ± 0.3	5.7 ± 1.2	−0.6 ± 1.2	0.0399	0.0929	−0.65 ± 0.27	(−1.192, −0.108)	0.0199*
	SlimSEB^®^	6.3 ± 0.3	5.1 ± 0.9	−1.2 ± 0.8	<0.001				
HOMA-IR	Placebo	3.78 ± 1.44	3.44 ± 1.62	−0.34 ± 0.59	0.0192	0.0144	−0.47 ± 0.2	(−0.873, −0.075)	0.0205*
	SlimSEB^®^	3.67 ± 1.57	2.88 ± 1.39	−0.79 ± 0.57	<0.0001				
HOMA-β	Placebo	86.88 ± 30.98	110.02 ± 53.86	23.14 ± 51.70	0.0598	0.3057	17.17 ± 12.63	(−8.11, 42.443)	0.1793
	SlimSEB^®^	82.68 ± 32.29	121.87 ± 67.18	39.19 ± 49.27	0.0010				
Disposition Index	Placebo	23.3 ± 2.4	37.0 ± 21.5	13.7 ± 22.4	0.0129	0.313	7 ± 4.98	(−2.94, 16.94)	0.1645
	SlimSEB^®^	23.0 ± 2.6	43.3 ± 18.6	20.3 ± 18.9	<0.0001				
QUICKI	Placebo	0.318 ± 0.018	0.324 ± 0.023	0.006 ± 0.00	0.007	0.052	0.0066 ± 0.0032	(0.0002, 0.0129)	0.0437
	SlimSEB^®^	0.321 ± 0.023	0.333 ± 0.025	0.012 ± 0.00	<0.001				
3. Lipid profile and biomarkers									
Total cholesterol (mg/dL)	Placebo	182 ± 18	175 ± 28	−8 ± 31	0.2713	0.1041	−20.54 ± 7.54	(−35.699, −5.38)	0.0089
	SlimSEB^®^	183 ± 31	157 ± 33	−26 ± 40	0.0053				
LDL (mg/dL)	Placebo	113 ± 21	112 ± 23	−1 ± 30	0.8476	0.2455	−14.86 ± 5.97	(−26.781, −2.942)	0.0154
	SlimSEB^®^	112 ± 22	99 ± 24	−13 ± 35	0.0856				
HDL (mg/dL)	Placebo	45 ± 7	45 ± 5	0 ± 5	0.9484	0.3884	0.46 ± 1.15	(−1.849, 2.766)	0.6919
	SlimSEB^®^	43 ± 6	44 ± 6	1 ± 5	0.2077				
Triglycerides (mg/dL)	Placebo	139 ± 25	142 ± 23	3 ± 25	0.5510	0.0194	−9.02 ± 7.07	(−23.132, 5.101)	0.2068
	SlimSEB^®^	150 ± 18	135 ± 19	−15 ± 23	0.0057				
CRP (mg/dL)	Placebo	1.94 ± 1.54	1.67 ± 1.28	−0.27 ± 0.70	0.0967	0.8883	0 ± 0.14	(−0.279, 0.272)	0.9793
	SlimSEB^®^	1.84 ± 1.36	1.59 ± 1.09	−0.25 ± 0.50	0.0264				
Obese subjects									
1. Anthropometric parameters									
Body weight (kg)	Placebo	86.2 ± 7.0	87.1 ± 6.6	0.9 ± 3.6	0.2419	<0.0001	−4.51 ± 0.74	(−5.984, −3.031)	<0.0001*
	SlimSEB^®^	86.5 ± 7.4	82.8 ± 7.2	−3.6 ± 1.7	<0.0001				
BMI (kg/m^2^)	Placebo	32.4 ± 1.9	32.8 ± 2.7	0.4 ± 1.4	0.1759	<0.0001	−1.8 ± 0.29	(−2.389, −1.22)	<0.0001*
	SlimSEB^®^	32.6 ± 1.9	31.2 ± 1.6	−1.4 ± 0.7	<0.0001				
Waist circumference (cm)	Placebo	104.2 ± 7.3	105.6 ± 8.9	1.4 ± 5.3	0.2302	0.0011	−11.03 ± 3.33	(−17.664, −4.402)	0.0014*
	SlimSEB^®^	104.4 ± 7.2	98.8 ± 8.2	−5.6 ± 7.4	0.0024				
2. Glucose and Insulin related factors									
Fasting plasma glucose (mmol/L)	Placebo	6.43 ± 0.21	6.02 ± 0.68	−0.42 ± 0.78	0.0180	0.0368	−0.45 ± 0.16	(−0.77, −0.129)	0.0068*
	SlimSEB^®^	6.46 ± 0.20	5.57 ± 0.58	−0.89 ± 0.68	<0.0001				
Fasting Insulin (μIU/mL)	Placebo	12.43 ± 4.11	11.93 ± 5.19	−0.50 ± 2.89	0.4154	0.2509	−1.78 ± 0.61	(−3.01, −0.55)	0.0054*
	SlimSEB^®^	12.94 ± 4.29	11.57 ± 3.82	−1.37 ± 2.03	0.0057				
HbA1c (%)	Placebo	6.3 ± 0.3	5.8 ± 0.5	−0.4 ± 0.8	0.0160	0.0047	−0.67 ± 0.22	(−1.11, −0.24)	0.003*
	SlimSEB^®^	6.4 ± 0.4	5.1 ± 1.0	−1.3 ± 1.1	<0.0001				
HOMA-IR	Placebo	3.48 ± 1.23	3.33 ± 1.38	−0.15 ± 1.01	0.4709	0.0061	−0.69 ± 0.21	(−1.12, −0.27)	0.002*
	SlimSEB^®^	3.71 ± 1.25	2.79 ± 1.04	−0.92 ± 0.74	<0.0001				
HOMA-β	Placebo	83.11 ± 27.85	111.14 ± 60.59	28.03 ± 47.72	0.0100	0.6502	4.44 ± 12.42	(−20.30, 29.18)	0.7217
	SlimSEB^®^	87.38 ± 29.13	121.88 ± 62.94	34.49 ± 46.04	0.0026				
Disposition Index	Placebo	24.0 ± 2.4	34.8 ± 19.3	10.1 ± 20.5	0.0192	0.120	9.86 ± 4.87	(0.15, 19.57)	0.0467*
	SlimSEB^®^	23.7 ± 2.3	44.8 ± 21.5	21.1 ± 22.4	0.0001				
QUICKI	Placebo	0.321 ± 0.019	0.312 ± 0.062	−0.009 ± 0.001	0.465	0.082	0.021 ± 0.0096	(0.002, 0.0401)	0.0317
	SlimSEB^®^	0.318 ± 0.018	0.332 ± 0.019	0.014 ± 0.001	<0.001				
3. Lipid profile and biomarkers									
Total cholesterol (mg/dL)	Placebo	185 ± 27	201 ± 25	16 ± 22	0.0024	<0.0001	−38.09 ± 6.72	(−51.547, −24.63)	<0.0001
	SlimSEB^®^	200 ± 30	168 ± 27	−32 ± 40	0.0015				
LDL (mg/dL)	Placebo	112 ± 29	127 ± 26	15 ± 23	0.0047	0.0006	−24.04 ± 6.13	(−36.286, −11.796)	0.0002
	SlimSEB^®^	123 ± 25	105 ± 20	−18 ± 34	0.0239				
HDL (mg/dL)	Placebo	46 ± 7	46 ± 8	−0.0 ± 8	0.9459	0.2700	0.85 ± 1.52	(−2.179, 3.884)	0.5758
	SlimSEB^®^	43 ± 6	45 ± 6	2 ± 4	0.0378				
Triglycerides (mg/dL)	Placebo	159 ± 21	156 ± 29	−3 ± 26	0.5926	0.1142	−13.75 ± 7.16	(−28.054, 0.561)	0.0594
	SlimSEB^®^	157 ± 18	142 ± 21	−15 ± 22	0.0060				
CRP (mg/dL)	Placebo	1.80 ± 1.48	1.91 ± 1.58	0.11 ± 0.35	0.1591	0.0044	−0.48 ± 0.12	(−0.71, −0.241)	0.0001
	SlimSEB^®^	2.20 ± 1.84	1.82 ± 1.41	−0.38 ± 0.65	0.0132				

*Intra group statistical analysis performed with “*t*-test: paired two sample for means”; **Inter-group statistical analysis was performed with “Independent *t*-tests assuming unequal variances.” ^$^Inter group statistical analysis was performed using “ANCOVA model with change from baseline as the dependent variable and visit, treatment, baseline variables such as diet, physical activity/excersie and treatment by visit interaction as a fixed effect and baseline score as a covariate. CI, confidence interval; SOT, Start of Treatment or baseline; EOT, End of Treatment; ‘EOT-SOT’ is the mean difference in values.

A similar trend of decline in BMI, with significant inter-group differences in both overweight and obese subjects. BMI decreased by 0.9 ± 1.5 kg/m^2^ with SlimSEB^®^ as compared to 0.1 ± 1.2 kg/m^2^, resulting in a statistically significant inter-group difference (*p* = 0.0454). ANCOVA confirmed this difference, with an adjusted LS mean difference of −0.92 kg/m^2^ (95% CI: −1.569, −0.275; *p* = 0.0063). Likewise, BMI decreased by 1.4 ± 0.7 kg/m^2^ in the SlimSEB^®^ compared with an increase of 0.4 ± 1.4 kg/m^2^ in the placebo group, yielding a statistically significant inter-group difference (*p* < 0.0001). The ANCOVA analysis confirmed this finding, with an adjusted LS mean difference of −1.8 kg/m^2^ (95% CI: −2.389, −1.22; *p* < 0.0001). The intra-group reductions were significant with SlimSEB^®^ in overweight (28.0 ± 0.9 [SOT] to 27.1 ± 2.0 kg/m^2^ [EOT]; *p* = 0.0078) and obese subjects (32.6 ± 1.9 [SOT] to 31.2 ± 1.6 kg/m^2^ [EOT]; *p* < 0.0001), while placebo showed no significant changes (*p* = 0.7757 and 0.1759), respectively.

Further, for the waist circumference, the reduction was greater with SlimSEB^®^ over the placebo in both overweight and obese subjects. In overweight subjects, the SlimSEB^®^ showed a greater reduction compared with placebo (−4.7 ± 6.6 vs. -1.2 ± 6.3 cm), with a statistically significant adjusted inter-group difference by ANCOVA (LS mean difference: −3.86 cm; 95% CI: −6.995, −0.726; *p* = 0.0169). The independent *t*-test comparison of mean change, however, did not reach statistical significance (*p* = 0.0777). In obese subjects, the inter-group difference was statistically significant based on the independent *t*-test (*p* = 0.0011). ANCOVA also demonstrated a statistically significant adjusted inter-group difference (−5.6 ± 7.4 cm vs. an increase of 1.4 ± 5.3 cm), with an LS mean difference of −11.03 cm (95% CI: −17.66, −4.402; *p* = 0.0014). The intra-group reductions with SlimSEB^®^ were significant in both overweight and obese subjects (*p* = 0.0024 for each), whereas no significant changes occurred with placebo (98.8 ± 7.8 cm [SOT] to 94.1 ± 10.6 cm [EOT]; *p* = 0.4246, and 104.4 ± 7.2 cm [SOT] to 98.8 ± 8.2 cm [EOT]; *p* = 0.2302, respectively).

#### 3.2.2 Effects of SlimSEB^®^ supplementation on the glucose and insulin related factors

Glucose and related insulin parameters were assessed before and after the intervention period (Table 3). Fasting plasma glucose 13.78% (0.96 ± 0.52 mmol/L) and 14.72% (0.89 ± 0.68 mmol/L), respectively, compared with reductions of 8% (0.52 ± 0.86 mmol/L) and 6.37% (0.42 ± 0.78 mmol/L) with placebo. SlimSEB^®^ resulted in a statistically significant inter-group difference (*p* = 0.0368) in obese subjects, but did not reach statistical significance by independent *t*-test (*p* = 0.0555) in overweight subjects. ANCOVA demonstrated a statistically significant adjusted inter-group difference, with an LS mean difference of −0.45 mmol/L (95% CI: −0.804, −0.102; *p* = 0.0124) in overweight and −0.45 mmol/L (95% CI: −0.77, −0.129; *p* = 0.0068) in obese subjects, respectively. The intra-group reductions were significant with both SlimSEB^®^ (*p* < 0.0001 for both) and placebo (*p* = 0.0144 and 0.0180) in overweight and obese subjects, respectively.

HbA1c decreased by 1.2 ± 0.8% and 1.3 ± 1.1% with SlimSEB^®^ in overweight and obese subjects, respectively, compared with reductions of 0.6 ± 1.2% and 0.4 ± 0.8% with placebo. The independent *t*-test demonstrated a statistically significant inter-group difference (*p* = 0.0047) in obese subjects, but failed to cause a statistically significant inter-group difference (*p* = 0.0929) in overweight subjects. However, ANCOVA showed a significant adjusted difference, with an LS mean difference of −0.65% (95% CI: −1.192, −0.108; *p* = 0.0199) and −0.67% (95% CI: −1.106, −0.24; *p* = 0.003) in overweight and obese subjects, respectively.

HOMA-IR decreased significantly with SlimSEB^®^ in both overweight (−0.79 ± 0.57) and obese subjects (−0.92 ± 0.74), compared with reductions of 0.34 ± 0.59 and 0.15 ± 1.01 with placebo, with a statistically significant inter-group difference in overweight (*p* = 0.0144) and obese (*p* = 0.0061) subjects. The ANCOVA analysis also confirmed a significant adjusted difference, with an LS mean difference of −0.47 (95% CI: −0.873, −0.075; *p* = 0.0205), and −0.69 (95% CI: −1.122, −0.266; *p* = 0.002) in overweight and obese subjects, respectively. The intra-group reductions were significant with SlimSEB^®^ (both *p* < 0.0001) and with placebo in overweight subjects (*p* = 0.0192), but not in obese subjects (*p* = 0.4709). In addition, all the other insulin related factors viz. fasting insulin, HOMA-β, DI and QUICKI had shown positive changes in their levels post intervention with the investigational products.

#### 3.2.3 Effects of SlimSEB^®^ supplementation on the lipid and inflammation markers

Lipid profile and inflammatory markers were performed to measure the lipids and CRP levels for monitoring and detecting changes in cardiovascular health and inflammation. The total cholesterol decreased by 32 ± 40 mg/dL with SlimSEB^®^, whereas it increased by 16 ± 22 mg/dL with placebo in obese subjects. The inter-group difference was highly statistically significant by independent *t*-test (*p* < 0.0001), with ANCOVA confirming a significant adjusted difference (LS mean difference: −38.09 mg/dL; 95% CI: −51.547, 24.63; *p* < 0.0001). In overweight subjects, the total cholesterol decreased by 26 ± 40 mg/dL with SlimSEB® compared with 8 ± 31 mg/dL with placebo. Although the independent *t*-test did not show a statistically significant inter-group difference (*p* = 0.1041), ANCOVA demonstrated a statistically significant adjusted difference, with an LS mean difference of −20.54 mg/dL (95% CI: −35.699, −5.38; *p* = 0.0089). Intra-group reductions were significant with SlimSEB^®^ in both overweight and obese subjects (*p* = 0.0053 and *p* = 0.0015), while placebo showed no significant change in overweight subjects (*p* = 0.2713) but a significant increase in obese subjects (*p* = 0.0024). The LDL levels decreased by 18 ± 34 mg/dL with SlimSEB^®^ compared with an increase of 15 ± 23 mg/dL with placebo in obese subjects, yielding a significant inter-group difference (*p* = 0.0006), and ANCOVA confirmed a significant adjusted difference (LS mean difference: −24.04 mg/dL; 95% CI: −36.29, −11.796; *p* = 0.0002). In overweight subjects, the LDL levels decreased by 13 ± 35 mg/dL with SlimSEB^®^ versus 1 ± 30 mg/dL with placebo, with no significant inter-group difference (*p* = 0.2455), however, ANCOVA demonstrated a significant adjusted inter-group difference (LS mean difference: −14.86 mg/dL; 95% CI: −26.781, −2.942; *p* = 0.0154). The intra-group changes showed significant changes in obese subjects (*p* = 0.0239 and *p* = 0.0047) for SlimSEB^®^ and placebo, respectively, whereas these were not significant in overweight subjects (*p* = 0.0856 and *p* = 0.8476). In addition, HDL and triglycerides in blood were also quantified, and showed positive changes from their initial levels post intervention with the SlimSEB^®^.

The changes in CRP were quantified due to its role in inflammation and cardiovascular disorders. CRP levels decreased by 0.25 ± 0.50 mg/dL with SlimSEB^®^ versus 0.27 ± 0.70 mg/dL with placebo in overweight subjects, with no significant inter-group difference by either the independent *t*-test (*p* = 0.8883), or ANCOVA (LS mean difference: 0.0 mg/dL; 95% CI: −0.28, 0.27; *p* = 0.9793). In obese subjects, CRP decreased by 0.38 ± 0.65 mg/dL with SlimSEB^®^ but increased by 0.11 ± 0.35 mg/dL with placebo, resulting in a significant inter-group difference (*p* = 0.0044), and ANCOVA demonstrated a highly significant adjusted difference, with an LS mean difference of −0.48 mg/dL (95% CI: −0.71, −0.241; *p* = 0.0001). Intra-group reduction by 13.58% (*p* = 0.0264) and 17.27% (*p =* 0.0132) was significant with SlimSEB^®^ in both overweight and obese subjects, whereas placebo showed no significant change (*p* = 0.0967 and *p* = 0.1591).

### 3.3 Secondary endpoint: safety evaluation

#### 3.3.1 Assessment of IP tolerance, adverse events, serious adverse events, and systemic biomarkers

The effect of oral supplementation of SlimSEB^®^ and maltodextrin on the secondary endpoints was assessed to evaluate the safety profile of the product/s on the study subjects. Safety was evaluated by assessing the tolerance, AEs, SAE.s, physical and haematological parameters, and biochemical biomarkers. Any symptoms and/or side effect/s that occurred during the study was documented for each subject, and scored according to the severity. SlimSEB^®^ was well tolerated by all the subjects in the clinical study throughout the study period (Table 4). SlimSEB^®^ had 100 and 95.7% very good tolerability in obese and overweight subjects, while 4.30% good tolerability in overweight subjects, respectively. Both, the enrolled subjects and the accompanying physician, reported zero or no reports of incidences related to any SAEs, and/or drastic change in systemic biomarkers during the intervention. Four subjects in overweight group had a total of four AEs and TEAEs (1-cold, 1-cough, 1-fever, 1-headache), while two subjects in obese group had four AEs and TEAEs (2-dry cough and 2-headache) post supplementation of SlimSEB^®^, respectively, and the severity of the events was mild, and showed complete recovery after concomitant therapy. None of the AEs judged by the medical professional, exhibited an association or correlation with the investigational product, further indicating the safety of SlimSEB^®^ for oral consumption.

**Table 4:** Effect of SlimSEB^®^ and placebo supplementation on tolerability, AEs and SAEs in overweight and obese subjects.

Parameters	Categories	Overweight subjects		Obese subjects	
		Placebo	SlimSEB^®^	Placebo	SlimSEB^®^
Tolerability	Very good	20 (100%)	22 (95.7%)	23 (100%)	21 (100%)
	Good	00	01 (4.30%)	00	00
AEs/SAEs	Any AEs	0 (0.0%)	4 (17.4%)	0 (0.0%)	2 (9.5%)
	Any SAEs	0 (0.0%)	0 (0.0%)	0 (0.0%)	0 (0.0%)
	Any TEAEs	0 (0.0%)	4 (17.4%)	0 (0.0%)	2 (9.5%)
	Dropped due to this TEAEs	0 (0.0%)	0 (0.0%)	0 (0.0%)	0 (0.0%)
	Severe TEAEs	0 (0.0%)	0 (0.0%)	0 (0.0%)	0 (0.0%)
	TEAEs Leading to Death	0 (0.0%)	0 (0.0%)	0 (0.0%)	0 (0.0%)
	TEAEs ongoing	0 (0.0%)	0 (0.0%)	0 (0.0%)	0 (0.0%)

The vital physical indicators which included body temperature, systolic blood pressure, diastolic blood pressure, pulse rate, and respiration rate were assessed by the principal investigator at each visit (Table 5). Neither the IP nor the placebo showed any effect on any of these vital indicators (statistically non-significant, *p* ≥ 0.05) during each visit to the site. The parameters obtained were in the normal working range for overweight and obese subjects in placebo and SlimSEB^®^ group. Additionally, the vital indicators associated with haematology and biochemistry were also determined at the SOT and EOT in both the groups for all subjects. The vital haematological indicators consisted of enumeration of complete and differential blood count, platelets (hemoglobin, red blood cell, mean cell volume, mean cell hemoglobin, mean cell hemoglobin concentration, white blood cell and platelet count), and the vital biochemical indicators included the measurement of ALT, AST, serum creatinine, BUN, total albumin and globulin (Table 6). The values of vital parameters in haematological and biochemical indicators were found to be within the standard regime of the reference values. The changes that occurred in the parameters of placebo arm and test arm at EOT and SOT were not altered and statistically non-significant (*p* ≥ 0.05).

**Table 5:** Effect of SlimSEB^®^ supplementation on vital physical indicators in overweight and obese subjects.

Parameter	Intervention Group	SOT	EOT	EOT-SOT	*p*-value (Intra group)*	*p*-value (Inter group; independent *t*-test)**	95% CI for mean difference
Overweight subjects							
Respiration rate (Breathes per min)	Placebo	16.7 ± 2.3	16.7 ± 2.1	−0.0 ± 2	0.923	0.789	[−1.12, 1.02]
	SlimSEB^®^	17.0 ± 2.2	17.1 ± 1.6	0.1 ± 2.1	0.766		[−0.77, 1.03]
Systolic blood pressure (mmHg)	Placebo	126 ± 5	124 ± 7	−2 ± 8	0.307	0.246	[−6.14, 2.04]
	SlimSEB^®^	127 ± 6	122 ± 6	−5 ± 9	0.010		[−9.06, −1.38]
Diastolic blood pressure (mmHG)	Placebo	90 ± 6	83 ± 6	−7 ± 9	0.001	0.140	[−11.75, −3.35]
	SlimSEB^®^	84 ± 4	81 ± 5	−4 ± 8	0.043		[−7.09, −0.12]
Pulse rate (Beats per min)	Placebo	84 ± 6	85 ± 5	1 ± 7	0.433	0.004	[−2.18, 4.88]
	SlimSEB^®^	88 ± 6	82 ± 5	−7 ± 10	0.003		[−10.71, −2.42]
Body temperature (°F)	Placebo	98.2 ± 0.3	98.3 ± 0.3	0.1 ± 0.4	0.172	0.615	[−0.07, 0.35]
	SlimSEB^®^	98.1 ± 0.3	98.2 ± 0.2	0.1 ± 0.3	0.283		[−0.07, 0.23]
Obese subjects							
Respiration rate (Breathes per min)	Placebo	16.1 ± 2.1	17.0 ± 1.9	0.9 ± 1.4	0.005	0.464	[0.30, 1.52]
	SlimSEB^®^	17.3 ± 2.7	16.7 ± 2.3	−0.6 ± 2.1	0.189		[−1.57, 0.33]
Systolic blood pressure (mmHg)	Placebo	122 ± 6	126 ± 4	5 ± 7	0.005	0.0002	[1.59, 7.89]
	SlimSEB^®^	128 ± 5	122 ± 6	−6 ± 9	0.012		[−10.05, −1.38]
Diastolic blood pressure (mmHG)	Placebo	87 ± 6	84 ± 3	−3 ± 8	0.057	0.359	[−6.72, 0.11]
	SlimSEB^®^	86 ± 5	80 ± 6	−6 ± 9	0.01		[−9.90, −1.52]
Pulse rate (Beats per min)	Placebo	92 ± 3	91 ± 5	−1 ± 4	0.356	0.008	[−2.78, 1.04]
	SlimSEB^®^	90 ± 2	93 ± 2	2 ± 3	0.001		[0.97, 3.41]
Body temperature (°F)	Placebo	98.2 ± 0.4	97.9 ± 0.5	−0.2 ± 0.7	0.114	0.157	[−0.56, 0.06]
	SlimSEB^®^	98.3 ± 0.3	97.8 ± 0.6	−0.6 ± 0.7	0.001		[−0.86, −0.24]

*Intra group statistical analysis performed with “*t*-test: paired two sample for means”; **Inter-group statistical analysis was performed with “Independent *t*-tests assuming unequal variances.” CI, confidence interval; SOT, start of treatment or baseline; EOT, end of treatment; ‘EOT-SOT’ is the mean difference in values.

**Table 6:** Effect of SlimSEB^®^ supplementation on haematological parameters and vital biochemical indicators in overweight and obese subjects.

Parameters	Intervention Group	SOT	EOT	EOT-SOT	*p*-value (Intra group)*	*p*-value (Inter group; independent *t*-test)**	95% CI for mean difference
Overweight subjects							
RBC (mill/mm^3^)	Placebo	4.9 ± 0.3	4.9 ± 0.3	0.0 ± 0.2	0.9918	0.1852	[−0.10, 0.10]
	SlimSEB^®^	4.7 ± 0.4	4.8 ± 0.3	0.1 ± 0.2	0.0701		[−0.01, 0.19]
Platelet (thou/mm^3^)	Placebo	285.8 ± 76.0	310.7 ± 71.3	24.9 ± 41.4	0.0146	0.5482	[5.50, 44.20]
	SlimSEB^®^	269.5 ± 55.9	286.3 ± 61.4	17.3 ± 40.0	0.0499		[0.01, 34.61]
Haemoglobin (g/dL)	Placebo	13.9 ± 1.8	14.3 ± 1.6	0.4 ± 1.0	0.0606	0.5313	[−0.02, 0.88]
	SlimSEB^®^	14.4 ± 2.0	14.4 ± 2.2	0.1 ± 1.7	0.8739		[−0.68, 0.79]
Haematocrit (%)	Placebo	41.0 ± 4.8	41.0 ± 3.6	0.0 ± 3.0	0.9584	0.2731	[−1.42, 1.35]
	SlimSEB^®^	41.7 ± 5.6	42.5 ± 5.0	0.8 ± 1.4	0.0142		[0.17, 1.38]
WBC (thou/mm^3^)	Placebo	7,872 ± 1665.8	7576.2 ± 1777.7	−295.8 ± 792.4	0.1114	0.3623	[−666.64, 75.04]
	SlimSEB^®^	7922.2 ± 1803.7	7817.0 ± 1693.6	−105.2 ± 504.9	0.3285		[−323.57, 113.14]
Neutrophils (%)	Placebo	63.0 ± 5.6	64.8 ± 6.0	1.8 ± 6.5	0.2280	0.2692	[−1.22, 4.82]
	SlimSEB^®^	60.5 ± 7.7	60.0 ± 7.3	−0.5 ± 7.1	0.7302		[−3.59, 2.55]
Lymphocytes (%)	Placebo	27 ± 4.4	26.2 ± 5.9	−0.9 ± 7.0	0.5917	0.1777	[−4.11, 2.41]
	SlimSEB^®^	28.4 ± 5.6	30.3 ± 6.0	1.9 ± 6.1	0.1471		[−0.73, 4.55]
Monocytes (%)	Placebo	6.1 ± 25.1	5.2 ± 2.6	−1.0 ± 2.2	0.0671	0.8260	[−1.97, 0.07]
	SlimSEB^®^	6.0 ± 2.9	5.2 ± 1.8	−0.8 ± 2.6	0.1672		[−1.93, 0.36]
Eosinophils (%)	Placebo	3.7 ± 1.5	3.6 ± 1.2	−0.1 ± 1.5	0.7663	0.9091	[−0.79, 0.59]
	SlimSEB^®^	4.3 ± 2.4	4.1 ± 1.6	−0.2 ± 2.6	0.7550		[−1.32, 0.97]
Basophils (%)	Placebo	0.3 ± 0.4	0.4 ± 0.5	0.1 ± 0. 5	0.3299	0.4592	[−0.11, 0.31]
	SlimSEB^®^	0.3 ± 0.5	0.3 ± 0.5	0.0 ± 0.4	1.0000		[−0.18, 0.18]
BUN (mg/dL)	Placebo	20.3 ± 7.7	19.8 ± 8.6	−0.5 ± 2.5	0.4363	0.6492	[−1.64, 0.74]
	SlimSEB^®^	24.0 ± 10.0	23.9 ± 10.7	−0.1 ± 2.9	0.9108		[−1.33, 1.20]
Serum creatinine (mg/dL)	Placebo	1.1 ± 0.2	1.1 ± 0.2	0.0 ± 0.1	0.9166	0.4477	[−0.06, 0.06]
	SlimSEB^®^	1.1 ± 0.2	1.1 ± 0.2	0.0 ± 0.1	0.2230		[−0.09, 0.02]
Albumin (g/dL)	Placebo	4.5 ± 0.8	4.5 ± 0.6	0.1 ± 0.6	0.5763	0.9692	[−0.21, 0.36]
	SlimSEB^®^	4.1 ± 0.3	4.2 ± 0.3	0.1 ± 0.2	0.0456		[0.00, 0.16]
Globulin (g/dL)	Placebo	2.7 ± 0.6	2.7 ± 0.5	0.0 ± 0.3	0.6280	0.6239	[−0.19, 0.12]
	SlimSEB^®^	2.9 ± 0.3	2.9 ± 0.5	0.0 ± 0.5	0.8080		[−0.18, 0.23]
ALT (U/L)	Placebo	27.4 ± 4.4	27.3 ± 4.6	−0.1 ± 4.9	0.9436	0.0991	[−2.39, 2.23]
	SlimSEB^®^	28.2 ± 6.2	25.7 ± 5.8	−2.5 ± 4.3	0.0114		[−4.35, −0.62]
AST (U/L)	Placebo	28.7 ± 5.2	26.9 ± 4.5	−1.9 ± 3.9	0.0466	0.2678	[−3.72, −0.03]
	SlimSEB^®^	28.7 ± 5.3	25.5 ± 5.0	−3.2 ± 3.6	0.0004		[−4.75, −1.61]
Obese subjects							
RBC (mill/mm^3^)	Placebo	4.8 ± 0.4	4.8 ± 0.3	0.0 ± 0.3	0.5621	0.33	[−0.19, 0.11,]
	SlimSEB^®^	4.7 ± 0.4	4.7 ± 0.3	0.0 ± 0.3	0.4071		[−0.07, 0.16]
Platelet (thou/mm^3^)	Placebo	263.3 ± 72.4	277 ± 76.6	13.7 ± 32.2	0.0535	0.4710	[−0.23, 27.60,]
	SlimSEB^®^	282.3 ± 50.3	303.4 ± 39.7	21.1 ± 34.7	0.0115		[5.26, 36.84,]
Haemoglobin (g/dL)	Placebo	14.4 ± 2.1	14.7 ± 1.7	0.3 ± 1.1	0.1567	0.6188	[−0.14, 0.81]
	SlimSEB^®^	14.3 ± 1.7	14.5 ± 1.5	0.1 ± 1.8	0.7849		[−0.71, 0.93]
Haematocrit (%)	Placebo	41.6 ± 6.2	42.5 ± 5.5	0.9 ± 2.9	0.1579	0.7826	[−0.37, 2.14]
	SlimSEB^®^	41.7 ± 5.3	42.4 ± 5.2	0.7 ± 1.8	0.0893		[−0.12, 1.49]
WBC (thou/mm^3^)	Placebo	8071.7 ± 2140.1	7749.8 ± 1774.5	−322.0 ± 770.6	0.0576	0.8665	[−655.20, 11.29]
	SlimSEB^®^	7905.7 ± 1523.7	7623.9 ± 1384.0	−281.9 ± 798.1	0.1213		[−645.15, 81.44]
Neutrophils (%)	Placebo	57.9 ± 7.6	57.3 ± 7.9	−0.6 ± 8.4	0.7311	0.2995	[−4.24, 3.02]
	SlimSEB^®^	61.9 ± 7.5	58.9 ± 7.5	−3.1 ± 7.0	0.0597		[−6.23, 0.14]
Lymphocytes (%)	Placebo	31.4 ± 6.4	31.9 ± 7.7	0.5 ± 7.4	0.7585	0.1302	[−2.71, 3.66,]
	SlimSEB^®^	28.0 ± 7.7	31.7 ± 7.6	3.8 ± 6.7	0.0188		[0.69, 6.83,]
Monocytes (%)	Placebo	6.0 ± 2.8	6.7 ± 2.7	0.7 ± 2.0	0.0978	0.0570	[−0.15, 1.63]
	SlimSEB^®^	6.3 ± 2.6	5.7 ± 2.8	−0.6 ± 2.4	0.2798		[−1.64, 0.50,]
Eosinophils (%)	Placebo	4.0 ± 1.8	4.0 ± 1.5	0.0 ± 1.8	0.9111	0.7362	[−0.75, 0.84]
	SlimSEB^®^	3.6 ± 1.8	3.4 ± 1.2	−0.2 ± 1.8	0.7194		[−0.96, 0.68]
Basophils (%)	Placebo	0.3 ± 0.5	0.2 ± 0.4	−0.1 ± 0.5	0.0829	0.2885	[−0.28, 0.02]
	SlimSEB^®^	0.3 ± 0.5	0.3 ± 0.5	0 ± 0.4	1.0000		[−0.20, 0.20]
BUN (mg/dL)	Placebo	21.5 ± 8.9	20.0 ± 9.3	−1.4 ± 2.6	0.0149	0.1372	[−2.58, −0.31]
	SlimSEB^®^	22.0 ± 8.7	21.5 ± 9.0	−0.5 ± 1. 4	0.1399		[−1.14, 0.17]
Serum creatinine (mg/dL)	Placebo	1.1 ± 0.2	1.1 ± 0.2	0.0 ± 0.10	0.7456	0.0736	[−0.05, 0.03]
	SlimSEB^®^	1.1 ± 0.2	1.0 ± 0.2	−0.1 ± 0.2	0.0367		[−0.17, −0.01]
Albumin (g/dL)	Placebo	4.3 ± 0.5	4.4 ± 0.8	0.1 ± 0.7	0.3506	0.3108	[−0.16, 0.44]
	SlimSEB^®^	4.2 ± 0.5	4.2 ± 0.5	0.0 ± 0.4	0.6900		[−0.21, 0.14]
Globulin (g/dL)	Placebo	3.1 ± 0.4	3.0 ± 0.4	−0.1 ± 0.3	0.3152	0.1570	[−0.19, 0.06]
	SlimSEB^®^	2.8 ± 0.4	2.9 ± 0.5	0.1 ± 0.4	0.3052		[−0.09, 0.28]
ALT (U/L)	Placebo	28.8 ± 6.2	27.3 ± 4.7	−1.5 ± 5.1	0.1726	0.6293	[−3.69, 0.70]
	SlimSEB^®^	28.6 ± 4.9	27.9 ± 5.0	−0.7 ± 5.4	0.5455		[−3.17, 1.73]
AST (U/L)	Placebo	30.0 ± 4.7	27.0 ± 5.4	−3.0 ± 4.9	0.0081	0.6453	[−5.12, −0.86]
	SlimSEB^®^	31.0 ± 5.6	27.4 ± 4.9	−3.6 ± 4.3	0.0011		[−5.62, −1.66]

*Intra group statistical analysis performed with “*t*-test: paired two sample for means”; **Inter-group statistical analysis was performed with “Independent *t*-tests assuming unequal variances.” CI, confidence interval; SOT, start of treatment or baseline; EOT, end of treatment; ‘EOT-SOT’ is the mean difference in values.

## 4 Discussion

In the current prospective, interventional, randomized, double-blind, parallel, and placebo controlled clinical investigation, overweight and obese subjects with the risks of T2DM were orally supplemented with SlimSEB^®^ or placebo and assessed for its safety, therapeutic efficacy in weight reduction. Explicitly, the efficacy of the IP was assessed in overweight and obese subjects using a set of primary outcomes and secondary outcomes. The intervention period of 84 days was allotted to investigate the outcomes based on the previous clinical studies by Kaczmarczyk et al. (48), Sudha et al. (49), Chen et al. (50), and Crovesy et al. (51), as this duration may be good for observing positive effects on anthropometric parameters.

In the present clinical investigation, the recruited subjects were randomized into the SlimSEB^®^ intervention and placebo groups. Based on BMI, the subjects were further allocated to the overweight and obese groups, and assessed for changes in anthropometric, glucose and insulin related factors, along with lipid and inflammation markers, respectively. Body weight decreased statistically by 3.2% with SlimSEB^®^ compared with 0.1% with placebo in overweight subjects and by 4.2% compared with a 1.0% increase in obese subjects, with significant inter-group differences in both categories (*p* = 0.0316, and *p* < 0.001), and these finding were supported by ANCOVA (*p* = 0.0045, *p* = 0.0083). BMI showed a similar pattern, decreasing by 0.9 kg/m^2^ and 1.4 kg/m^2^ with SlimSEB^®^ compared with minimal change or an increase with placebo (*p* = 0.0454, and *p* < 0.001), and confirmed ANCOVA analysis (*p* = 0.0063, *p* < 0.0001). The inter-group difference in waist circumference was significant in obese subjects (*p* = 0.0011), where SlimSEB^®^ produced a 5.6 ± 7.4 cm reduction compared with a 1.4 ± 5.3 cm increase with placebo, but did not reach significance in overweight subjects. ANCOVA demonstrated a statistically significant adjusted inter-group difference with LS mean difference −3.86 cm (*p* = 0.0169) and −11.03 cm (*p* = 0.0014) in overweight and obese subjcts. The observed changes in anthropometric parameters are in agreement with evidence from a pilot study investigating SlimSEB^®^ supplementation in GLP-1-treated patients with T2DM, where weight reduction was reported (44). A recent systematic review has investigated the effect of probiotics and synbiotics on weight reduction of overweight and obese subjects, and documented similar findings (51, 52).

The metabolic findings were particularly notable for insulin resistance. HOMA-IR decreased by 21.5% with SlimSEB^®^ versus 9.0% with placebo in overweight subjects and by 24.8% versus 4.3%, respectively, in obese subjects. The significant inter-group differences in both categories (*p* = 0.0144, and *p* = 0.0061) provide stronger evidence for an effect on insulin resistance than the within-group changes in individual insulin-related parameters. IFG is defined as 100–125 mg/dL, but in the current study, the subjects with an IFG levels of 110–125 mg/dL were included to minimize the error for accidental inclusion of subjects with normal IFG levels. The fasting plasma glucose levels showed a greater reduction with SlimSEB^®^ in both groups; however, the inter-group difference reached statistical significance only in obese subjects (*p* = 0.0368). The ANCOVA demonstrated a statistically significant adjusted inter-group difference (*p* = 0.0124) in overweight and (*p* = 0.0068) in obese subjects, respectively. Similarly, HbA1c decreased substantially with SlimSEB^®^ in both weight categories, but independent *t*-test demonstrated a statistically significant inter-group difference (*p* = 0.0047) in obese subjects, and not in overweight subjects (*p* = 0.0929). However, ANCOVA showed a significant statistical significance in both weight categories with an LS mean difference of −0.65% (*p* = 0.0199) and −0.67% (*p* = 0.003). These findings suggest that the metabolic response was more clearly differentiated from placebo in subjects with obesity. The remaining insulin-related parameters showed a less consistent inter-group profile. Although fasting insulin, HOMA-β, Disposition Index, and QUICKI generally improved with SlimSEB^®^, their inter-group differences were not statistically significant (*p* ≥ 0.05). Therefore, these outcomes provide supportive evidence of metabolic changes but should not be interpreted as definitive treatmen*t*-specific effects.

The lipid profile also demonstrated parameter and weight category specific inter-group effects. In obese subjects, the clearest inter-group differences were observed for total cholesterol and LDL, both of which decreased with SlimSEB^®^ while increasing with placebo (*p* < 0.001, and *p* = 0.0006). HDL did not differ significantly between groups in either weight category. Whereas, it did not cause substantial major alterations in the other lipid parameters as compared to placebo (*p* ≥ 0.05). The variations in the findings of glucose and lipid profile could be ascribed to the fact that no specific exercise schedule, or dietary plan nor restriction was followed, and in-line results were documented by Sudha et al. (49). A similar pattern was observed for CRP. Although CRP decreased with SlimSEB^®^ in both overweight and obese subjects, the inter-group difference was significant only in obese subjects, where CRP decreased by approximately 17.3% compared with a 6.1% increase with placebo (*p* = 0.0044), and non-significantly in overweight subjects by 13.6% compared to 13.92% (*p* = 0.8883). This indicated a steep reduction in the inflammation levels in the body that could have been caused by the weight related issues.

The probiotics, digestive enzymes, and herbal extracts may contribute to the regulation of metabolism of sugars, proteins and lipids, functioning of liver and other organs, preservation of mineral and vitamin levels, and supporting vital metabolic functions. The probiotics present in SlimSEB^®^ may colonize the intestine and produce lactic acids, SCFAs, and other secondary metabolites like enzymes, anti-microbial peptides, mucin peptides, and provide their beneficial effects (26). They may also help restore the gut microbiota to a beneficial microbial population, replenish useful functional activities, and support recovery and integrity of the intestinal mucosa (28). Probiotics may contribute to intestinal pH regulation, maintenance of intestinal homeostasis, and attenuation of gut microbiota dysbiosis, potentially accompanied by increased production of SCFAs. These SCFAs may influence energy homeostasis and adipose tissue metabolism through multiple mechanisms (27). They may promote thermogenesis by upregulating the expression of peroxisome proliferator activated receptor gamma coactivator-1a, and adenosine monophosphate-activated protein kinase in the muscle and liver tissues, thereby enhancing fatty acid oxidation. Additionally, SCFAs may induce thermogenesis in brown adipose tissue by promoting the browning of white adipose tissue and upregulating mitochondrial uncoupling protein-1. SCFAs may also paly role in regulation of energy homeostasis by binding to the G protein-coupled receptors on intestinal epithelial cells, and causing enhanced release of gut hormones such as glucagon-like peptides 1 and 2, and peptide YY. These hormones could have prominent roles in enhancing satiety, gastric emptying, and reducing fat mass (53–56).

The probiotics may affect the glucose metabolism by modulating the glucose homeostasis, insulin secretion and sensitivity, and impacting glucose absorption and utilization (57). Probiotics could help promote glucose-induced insulin secretion, and might eventually help reduce body weight and also secrete inflammatory mediators (58). These gut derived metabolites may be transported to liver via the portal vein, which directly affects its function and influences its susceptibility to metabolic changes (59). The probiotics have displayed positive roles in reducing the size of adipocytes by improving the expression of genes involved in the oxidation of fatty acids, and decreasing the absorption of fatty acids (30). In addition, gut microbiota has displayed vital roles in positively modulating the bile acid metabolism and supporting the production of dehyrogenases which could affect the levels of plasma cholesterol by transforming them into poorly absorbed sterol coprostanol (60). This could be probably due to the action of bile acid hydrochloride hydrolases produced by some probiotics causing the deconjugation of bile acids in the intestinal lumen. Further, probiotics not only help in suppressing the intestinal absorption of cholesterol into the brush border cells, but also support the reduction of endogenous cholesterol synthesis by inhibiting HMG-CoA reductase enzyme (54). In addition, the weight-reducing effects of probiotics may be ascribed to through multiple mechanisms, including appetite suppression, increased secretion of anorexigenic hormones, regulation of energy homeostasis, and attenuation of systemic inflammation (53–55). Furthermore, modulation of the gut microbiome may promote lipid oxidation in skeletal muscles and the liver through increased activation of adenosine monophosphate-activated protein kinase (AMPK), while potentially reducing adipose tissue fat storage by altering fasting-induced adipose factor (FIAF) levels, an inhibitor of lipoprotein lipase activity (61).

The exogenous digestive enzymes may contribute to digestion of nutrients in food in synchronization with the endogenous enzymes and speedup the digestion to enhance the availability of nutrients for absorption (32). These enzymes may act in GI tract and have an indirect role in body fluids upon absorption in small intestine, to confer the related health benefits (33). SucroSEB™ plays a role in converting sucrose to dietary fibers in presence of acceptor sugars and thus reducing the levels of sugars in gastric phase of digestion (34). The dietary fibers formed could further play a positive role in supporting the growth of probiotics, and also exert other health benefits by enhancing gut motility, supporting weight management, reducing abdominal adiposity, help manage sugar and lipid profile, roles in reducing chronic inflammation, and many others (35). In addition, the transglucosidase (or glucosyltransferase) has shown to improve the gut microbiota profile in patients with T2DM (36). Lactase hydrolyses the β-D-1,4 glycosidic linkage of lactose, and produce its monomeric components (62).

Further, the products derived from herbs are prominent sources of vital phyto-ingredients, which confers anti-inflammatory, antioxidant, anti-microbial, immunomodulatory, blood pressure reducing, thrombotic, and anti-diabetic properties (42, 63). The herbal products support weight loss by either appetite suppressant action and reducing the energy intake, thermogenesis stimulation and promotion of metabolism, pancreatic lipase activity inhibition and fat absorption reduction, enhanced lipolysis, and/or lipogenesis inhibition (44). Recent studies have showcased the anti-obesity, cholesterol reducing, anti-inflammatory, antioxidant, blood pressure improving, thrombotic, and anti-diabetes properties of mulberry leaf extract (63) and green tea (64). The bioactive constituents of mulberry that may be contributing to these biological activities are γ-aminobutyric acid, rutin, chlorogenic acid, astragalin, isoquercitin, and 1-deoxynojirimycin (65). Similarly, green tea extract may potentially improve insulin sensitivity and possibly alter fat metabolism. It may also increase energy expenditure, and influencing metabolic and lipid-digestion processes, thereby contributing to the management of hyperglycemia and hyperlipidemia (66, 67). In addition, the probiotics are also known to reduce inflammation by lowering CRP, and pro-inflammatory signals (68). Collectively, these mechanisms may support the hypothesis that a combination of probiotics, digestive enzymes, fiber-forming enzymes, and bioactive herbal compounds could have beneficial effects on gastrointestinal function, gut microbiota, adiposity, and metabolic health.

In an investigation with oral supplementation of transglucosidase for 12-weeks, desired changes in blood glucose, HbA1c and insulin levels were seen (36). Further, positive alterations in gut microbiome, and modifications in bowel mobility, and short chain fatty acids profile of feces in subjects with T2DM were also reported (37, 38). In another clinical study, the orally supplemented lactase had significantly reduced the mean hydrogen breath levels, and caused a substantial decline in abdominal pain, bloating, flatulence, borborygmi and diarrhea (62). In addition, a study performed on overweight and obese subjects with oral supplementation of multi-strain probiotics (*L. salivarius* UBLS-22, *L. casei* UBLC-42, *L. plantarum* UBLP-40, *L. acidophilus* UBLA-34, *B. breve* UBBr-01, *B. coagulans* Unique IS2; at a dose 5 billion CFU each, and total dose of probiotic 30 billion CFU) was found effective in significantly reducing the BMI, waist to hip ratio, body weight, and visceral fat (49). In another clinical investigation, daily supplementation with a multi-strain probiotic formulation containing *B. bifidum* W23, *B. lactis* W51, *B. lactis* W52, *L. acidophilus* W37, *L. brevis* W63, *L. casei* W56, *L. salivarius* W24, *L. lactis* W19, and *L. lactis* W58 (2.5 billion CFU/day) for 12 weeks demonstrated an association between weight loss and changes in gut microbiota composition in postmenopausal women with obesity (48). Similarly, a considerable changes in HDL and adiponectin, along with positive reduction in BMI, serum LDL, leptin, total cholesterol, and TNF-α levels were reported after a 12-week intervention period in obese children with a probiotic mixture (total 10 billion/day) containing *L. salivarius* AP-32, *L. rhamnosus* bv-77, and *B. animalis* CP-9, along with diet and proper exercise (50). The clinical studies on probiotics, prebiotics and synbiotics for body weights in patients with diabetes, overweight or obesity are discussed precisely in a systematic reviews and meta-analyses of randomized-controlled trials (52, 69). Both these reviews have indicated potential roles of probiotics in significantly reducing the body weight, BMI and waist circumference. Evidently, few clinical studies have also reported the efficacy of digestive enzymes in sugar management. The supplementation of a product containing probiotics (*B. infantis*, *B. bifidum*, *L. acidophilus*, *L. salivarius*, *L. plantarum*, *L. rhamnosus*, *B. longum*, *L. casei*, *L. paracasei*), and digestive enzymes (amylase, glucoamylase, lipase, bromelain, maltase, hemicellulase, lactase, xylanase, papain, and invertase) in hypercholesterolemia mice for 8 weeks caused a significant reduction in LDL, and an increase in HDL, while the triglyceride concentrations remained unchanged. Whereas a significant increase in propionic acid in the colon indicated the microbial production of SCFAs that inhibits the cholesterol synthesis (70).

In the current clinical investigation, the safety assessment unveiled that SlimSEB^®^ was well tolerated by all the subjects under study, and eight mild AEs were reported in six participants, none of which was considered related to SlimSEB^®^, and no SAEs were reported. Safety was evaluated by examining the vital physical, haematological, and biochemical parameters pre and post intervention. The values of these parameters after the end of treatment and study were at similar levels as those at baselines, and the slight changes that occurred were statistically non-significant (*p* ≥ 0.05). These clinically relevant and vital outcomes potentially indicate the safety of the orally supplemented SlimSEB^®^ at the studied dose. Further, no AEs and SAEs related to SlimSEB^®^ were reported by the participants or the clinical physician during the intervention period and the follow-up period. The safety and tolerability of SlimSEB^®^ as an adjunct to GLP-1 therapy in patients with T2DM demonstrated a favorable safety and tolerability profile during the study period, with no clinically significant safety concerns or serious adverse events observed (44). The tolerance and safety of transglucosidase (or glucosyltransferease), lactase, digestive enzymes (protease, amylase, lipase, cellulase and lactase) is already proved in earlier clinical studies (26, 57, 71). In addition, the earlier studies on multi-strain containing nine probiotics 2.5 billion/day was found safe in postmenopausal women with obesity (48). Similarly, a clinical investigation with oral supplementation of multi-strain probiotic strains at a dose of 5 billion CFU each (total dose of probiotic 30 billion CFU) was found to be effective and safe in the management of weight in obese or overweight adults (49). The clinical safety and tolerance of mulberry leaf or its extract, and green tea extract has been already proven in earlier investigations and thoroughly explained in systematic review and meta-analysis of randomized clinical trials by Cui et al. (72) and Lin et al. (73).

The present study has several limitations. First, dietary intake and physical activity are important determinants of body weight and metabolic health; however, these factors were not monitored or controlled, representing a major limitation of the study. Second, although weight reduction was the primary outcome, additional parameters associated with weight management, including glucose and insulin related factors and lipid profile, were also evaluated.

## 5 Conclusion

This prospective, interventional, randomized, double-blind, parallel, and placebo controlled clinical investigation in overweight and obese subjects with the risks of T2DM sheds light and presents preliminary evidence that a combination of probiotics, enzymes, and herbal extract can positively support the reduction and management of weight, glycemic factors, cholesterol and other parameters that may contribute for overweight and obesity over the placebo. SlimSEB^®^, at the studied dose, was well tolerated by the study population under study and found to be safe. Eight AEs (none of which was considered related to SlimSEB^®^) and zero reports of SAEs, negligible utilization of emergency and/or rescue medications and treatment further established the safety and effectiveness of SlimSEB^®^. The findings in present clinical study are of vital importance to the subjects willing to manage and/or control their weight, glucose and lipid levels. Although the results of current clinical trial have shed light of significance of SlimSEB^®^ as a novel and alternate approach for management of weight and reducing blood glucose and lipid levels, there is a need of further clinical trials to warrant the effects and confirm the findings in larger study populations and thus elucidate the underlying mechanisms.

## Data Availability

The original contributions presented in the study are included in the article/supplementary material, further inquiries can be directed to the corresponding author.
